# Different Nitro-Oxidative Response of *Odontarrhena lesbiaca* Plants from Geographically Separated Habitats to Excess Nickel

**DOI:** 10.3390/antiox9090837

**Published:** 2020-09-07

**Authors:** Gábor Feigl, Viktória Varga, Árpád Molnár, Panayiotis G. Dimitrakopoulos, Zsuzsanna Kolbert

**Affiliations:** 1 Department of Plant Biology, University of Szeged, Közép fasor 52, H6726 Szeged, Hungary; v.viki0709@gmail.com (V.V.); molnara@bio.u-szeged.hu (Á.M.); kolzsu@bio.u-szeged.hu (Z.K.); 2 Department of Environment, University of the Aegean, 81100 Mytilene, Lesbos, Greece; pdimi@env.aegean.gr

**Keywords:** *Alyssum lesbiacum*, nickel, hyperaccumulator, nitric oxide, tyrosine nitration

## Abstract

*Odontarrhena lesbiaca* is an endemic species to the serpentine soils of Lesbos Island (Greece). As a nickel (Ni) hyperaccumulator, it possesses an exceptional Ni tolerance; and it can accumulate up to 0.2–2.4% Ni of its leaves’ dry weight. In our study, *O. lesbiaca* seeds from two geographically separated study sites (Ampeliko and Loutra) were germinated and grown on control and Ni-containing (3000 mg/kg) soil in a rhizotron system. Ni excess induced significant Ni uptake and translocation in both *O. lesbiaca* ecotypes and affected their root architecture differently: plants from the Ampeliko site proved to be more tolerant; since their root growth was less inhibited compared to plants originated from the Loutra site. In the roots of the Ampeliko ecotype nitric oxide (NO) was being accumulated, while the degree of protein tyrosine nitration decreased; suggesting that NO in this case acts as a signaling molecule. Moreover, the detected decrease in protein tyrosine nitration may serve as an indicator of this ecotype’s better relative tolerance compared to the more sensitive plants originated from Loutra. Results suggest that Ni hypertolerance and the ability of hyperaccumulation might be connected to the plants’ capability of maintaining their nitrosative balance; yet, relatively little is known about the relationship between excess Ni, tolerance mechanisms and the balance of reactive nitrogen species in plants so far.

## 1. Introduction

Nickel (Ni) is the fifth most abundant element on Earth [1], frequently used in the steel, galvanic and electric industry. The average Ni content of the soil is usually not higher than 100 mg/kg [2], but it can be increased by industrial and municipal wastewaters, mining, cement industry or by burning diesel oil [3,4]. In addition to anthropogenic causes, high Ni content occurs naturally as well: serpentine soils might contain several thousand mg/kg Ni. Serpentine soils are far from ideal for most plants: besides their granular-rocky texture, they also possess low water-holding capacity [5]. Moreover, according to the criteria of the “serpentine syndrome” the availability of calcium is relatively low to magnesium, such soils usually deficient in essential nutrients as phosphorous, nitrogen and potassium, while they contain excessive amount of toxic heavy metals such as, manganese, cobalt, chromium or nickel [6].

The harshness of the conditions for plant survival and growth in serpentine environments has led plant species to evolve adaptive mechanisms to living on those [7]. One of the most notable strategies of plants to cope with elevated soil metal concentrations of serpentine habitats is hyperaccumulation. Hyperaccumulation that has been reported for metals and metalloids such as Ni, Zn, Cd, Pb, Co, Cr, Cu, Mn, Se, As and Tl is a rather rare phenomenon, as it is referred to about 2% of plants growing on serpentine at the global level [7]. From the more than 700 hyperaccumulator vascular plant species, 61 belong to the *Odontarrhena* genus (syn. *Alyssum* sect. *Odontarrhena*) [8], mainly growing on serpentine soils in southeast Europe and in the Middle East [9], and 523 have been characterized as Ni hyperaccumulators worldwide [8], as they are able to actively accumulate more than 1000 mg/kg Ni in their shoots [10].

Hyperaccumulation can vary to a great extent among and within populations of a species [11], driven by an interaction between genotype and environment [12]. The ability of a species to uptake and store metals is genetically regulated, but the environmental context in which it lives matters [12], especially local soil conditions and features such as total soil concentration of the metal of interest, soil concentration of important elements, soil porosity and pH [13]. Hyperaccumulators are distinguished in facultative hyperaccumulators of which some populations are capable of hyperaccumulating metals when are present on metal-rich soils, but other populations that occur in non-metalliferous soils do not show this property [11,12], and in obligate hyperaccumulators, i.e., serpentine-endemic species that are always able to uptake metals in levels above the hyperaccumulation-defined threshold values [12,13].

The large portion of hyperaccumulating species (85–90%) are serpentine-endemic ones [14]. Studies on serpentine endemic hyperaccumulating species have been demonstrated genetically distinct populations, with a relatively high genetic heterogeneity within the same population, and an important effect of geographic isolation on the genetic differentiation of populations [15,16,17]. In addition, field studies showed populations with different hyperaccumulating capacity related to soil nickel levels in the habitat of their origin for various *Odontarrhena* species [16,18,19], demonstrating the influence of small-scale serpentine-soil heterogeneity in determining intraspecific variation in foliar nickel concentration [16,19], as well as in other plant functional traits [7].

Ni is an essential micronutrient for plants as it is crucial for the activity of many enzymes such as urease [20], hydrogenase or Ni-superoxide dismutase [2]. However, in non-hyperaccumulator species, excess Ni has a negative effect on plant growth and physiology. It has the ability to disrupt general metabolic processes, causing morphological alterations like chlorosis or necrosis on leaves or deformed root tips and decreased root growth (reviewed by [2]), but Ni is also known to be able to reduce cell walls’ plasticity [21]. The toxicity of Ni is often connected with its negative effect on ion balance and disruption of membranes’ functionality [22]. Membrane stability can be compromised by the overproduction of reactive oxygen species (ROS) through the process of lipid peroxidation [23]. Ni is able to induce the formation of ROS (including hydrogen peroxide and hydroxyl radical) and cause oxidative stress, however, due to its redox-inactivity, in an indirect way [24,25].

While Ni induced imbalance of the ROS homeostasis is known in a number of species [2,26,27,28], little is known about its effect on the homeostasis of reactive nitrogen species (RNS) [29]. Nitric oxide (NO) and its reaction products, like the non-radical peroxynitrite (ONOO^−^), are probably the two most important members of the RNS [30]. The signaling of ROS and RNS intersects at many areas, laying the foundation of the rising examination of nitro-oxidative stress on the field of plant biology [31]. One of the main impact points of the RNS is the covalent modification of proteins. During the process of protein tyrosine nitration, ONOO^−^ is formed from NO and superoxide radical, leading to the covalent addition of a nitro-group to specific tyrosines of the proteins, resulting in a modified structure and activity, which can be either mostly loss or sometimes gain of function, and also no obvious effect on proteins’ function is possible [32,33,34]. Protein tyrosine nitration is also able to alter another signal transduction pathway by preventing phosphorylation of tyrosines [35]. Heavy metals are reportedly able to induce protein tyrosine nitration. It was detected in soybean as the effect of cadmium [36], in *Brassica napus* and *B. juncea* in the presence of excess zinc [37,38], or in case of combined heavy metal exposure in *B. napus* and *Helianthus annuus* [39]. It was also induced by Ni in the non-hyperaccumulator *Arabidopsis thaliana* and *B. juncea* [29], but so far, we have no information regarding the nitro-oxidative response of any Ni hyperaccumulator plant species.

*Odontarrhena lesbiaca* P. Candargy (≡*Alyssum lesbiacum* (P. Candargy) Rech. f.), is a perennial, maximum 40 cm tall plant, growing exclusively on serpentine soils of Lesbos, Greece [19,40]. This species is a strong Ni hyperaccumulator [19,41]. *O. lesbiaca* populations from different sites diverge in their Ni hyperaccumulation capacity, depending on the amount of Ni in the soil in the original habitat [19]. In addition, Ni tolerance, accumulation and translocation capacity of the same populations were significantly differed under hydroponic conditions [42]. Chelation is one possible tolerance mechanism for high amount of Ni [43], and *O. lesbiaca* was shown to contain an elevated concentration of histidine in its roots, which presumably plays an important role in Ni tolerance and translocation [44]. As another possible tolerance mechanism (to deposit Ni in the vacuoles of aerial parts, where its presence is less dangerous), high ATPase-dependent Ni/proton antiport capacity in the leaf cells’ vacuoles was also reported in *O. lesbiaca* [45]. Since *O. lesbiaca* accumulates a high amount of Ni in its foliage, insects (true bugs and crickets) consuming leaves accumulate a high amount of Ni, while low Ni loads were detectable in floral-reward consumers [46].

The aim of the present study was to examine nitro-oxidative stress in the root system of the Ni hyperaccumulator *O. lesbiaca* grown in a soil filled rhizotron system. For this study, two geographically distant serpentine habitats on Lesbos were selected that were differed in their soil Ni content [19] and they support genetically different populations of *O. lesbiaca* [17]. Our goal was to determine how excess Ni modifies the root system architecture of *O. lesbiaca* originating from the two different habitats. To obtain this, differences in the Ni-induced changes in the root growth and development of the two ecotypes was studied in a soil filled rhizotron system, together with the underlying changes in the nitro-oxidative status of the roots.

## 2. Materials and Methods

### 2.1. Plant Growing Conditions

Two geographically distant serpentine sites were selected at the Lesbos Island, Greece: Loutra is located at the eastern part of the island while Ampeliko is located at the central part of the island (site details described in [19]). The soil of the Ampeliko site had a higher Ni content (3326 ± 202 ppm), relative to Loutra (1197 ± 16.8 ppm) [19]. Both sites supported large populations of *O. lesbiaca* [19]. Loutra population is genetically differentiated from the populations grown at the central part of the island in which Ampeliko population is included [17].

At the community level, *O. lesbiaca* was highly dominant contributing significantly to the aboveground standing biomass in each site ranging from about 50% in Loutra to more than 85% in Ambeliko [47]. Other co-occurring dominant species were *Plantago lagopus* L. and *Hordeum bulbosum* L. at Ampeliko, and *P. lagopus*, *Aegilops biuncialis* Vis. and *Crepis commutata* (Spreng.) Greuter at Loutra [47]. Total and standing aboveground biomass, species richness and Shannon diversity did not appear to have significant differences between the two sites [48].

*O. lesbiaca* seeds were collected from more than 100 randomly selected individuals in each of the two sites. Seeds were germinated on moist filter paper in darkness, at 26 °C for 72 h. Germinated seeds then were transferred to rhizotrons (one seedling per rhizotron; system described in detail in [49]) filled with a soil mixture prepared using Klasmann Potgrond P blocking substrate (100% frozen through black peat with a fine structure of maximum 8 mm size, pH 6.0; 210 mg/L N; 240 mg/L P_2_O_5_, 270 mg/L K_2_O) mixed with 20% sand. During the experiments, two different rhizotron setups were used: the effect of control soil was compared to soil containing 3000 ppm Ni. This applied amount of Ni was chosen after testing a range of Ni concentrations because it resulted in an approximately 50% root growth inhibition in the more sensitive Loutra ecotype (Figure S1). Initial water content was adjusted to 70%, then the rhizotrons were watered with 10 mL distilled water on every second days. Seedlings were grown in the rhizotron systems for 14 days in a greenhouse at photon flux density of 150 µmol/m^2^/s (12/12 h light/dark cycle) at a relative humidity of 55–60% and 25 ± 2°C. After 2 weeks, the rhizotrons were scanned and the roots were cleaned for further analysis.

### 2.2. Examination of the Root System Architecture

Scanned images were analyzed using Fiji software (http://fiji.sc/Fiji; [50]). In addition to the length of the primary root (PR; cm), the number (number per root) and the length of the visible lateral roots (LR; cm) were examined, together with their angles included with the PR (degrees).

### 2.3. Analysis of Ni Content

Prior to the measurement of the Ni content of the soil (initial and final value) 200 g of soil samples were dried (105 °C, 24 h) and digested in aqua regia (hydrochloric acid: nitric acid = 3:1) in a microwave oven (Anton Paar Multiwave 3000, Graz, Austria). Ni concentration was measured by inductively coupled plasma atomic/optical emission spectrometer (ICP-OES, Perkin Elmer Optima 7000DV, Waltham, MA, USA) [51], and given in ppm (equals µg/g dry weight (DW)). The Ni content of the plant organs (root and shoot) were measured with inductively-coupled plasma mass spectrometry (ICP-MS, Agilent 7700 Series, Santa Clara, CA, USA) as described by Lehotai et al. [52] and given in ppm (equals µg/g dry weight (DW)).

Bioaccumulation factor (BAF; ratio of Ni concentration in the plant to the soil) [53] and translocation factor for Ni (TF, Ni concentration in the shoot/root) [54] were calculated to assess the Ni uptake potential of the *O. lesbiaca* seedlings.

### 2.4. Determination of the Root Apical Meristem Viability

Viability (indicated by intracellular esterase activity and membrane integrity) of the root apical meristem cells were examined with fluorescein diacetate (FDA) staining, as described in Feigl et al. [49].

### 2.5. Detection of ROS and RNS

Fluorescence associated with the amount of superoxide anion was detected by dihydroethidium (DHE) staining. Root tips were incubated in darkness in 10 µM dye solution (prepared in 10 mM Tris/HCl buffer, pH 7.4) for 30 min at 37 °C and then washed twice with buffer before examination [55].

Fluorescence consistent with hydrogen peroxide was visualized with 10-acetyl-3,7-dihydroxyphenoxazine (ADHP, Amplex Red or Ampliflu^TM^; Carlsbad, CA, USA) staining. 50 µM dye solution was prepared in 50 mM sodium-phosphate buffer (pH 7.5), in which root tips were incubated for 30 min, followed by one was with the same buffer [52].

NO-dependent fluorescence was determined by 4-amino-5-methylamino-2′,7′-difluorofluorescein diacetate (DAF-FM DA) staining. Root tips were incubated in 10 µM dye solution (in 10 mM Tris/HCl buffer, pH 7.4) at room temperature for 30 min, then washed twice with the same buffer [56].

Fluorescence associated with ONOO^−^ content of the root tips was detected with 10 µM 3′-(p-aminophenyl) fluorescein (APF, prepared in 10 mM Tris/HCl buffer, pH 7.4). Root tips were incubated in the dye solution for one hour, followed by two washings with the same buffer [57].

### 2.6. Microscopy

After the staining procedure, root tips were examined under a Zeiss Axiovert 200M inverted microscope (Carl Zeiss, Jena, Germany). Filter set 9 was used for DHE stained samples (exc.: 450–490 nm, em.: 515–∞ nm), filter set 20HE for Amplex Red (exc.: 546/12, em.: 607/80), while filter set 10 for FDA, DAF-FM DA and APF (exc.: 450–490, em.: 515–565 nm). On the obtained photos, fluorescence intensities (pixel intensity, proportional to the amount of the detected molecule) was measured in the root tips’ meristematic zone within circles of 50 µm radii using Axiovision Rel. 4.8 software (Carl Zeiss Microscopy GmbH, Oberkochen, Germany). In each case at least 10 samples were measured.

### 2.7. Western Blot Detection of Nitrated Proteins

Prior to the detection of protein tyrosine nitration, protein extracts were prepared from the root tissues according to Kolbert et al. [58], and their concentration was measured with Bradford assay [59] with using bovine serum albumin as standard. After that, 20 µL of root protein extracts per lane were separated on 12% acrylamide gels (sodium dodecyl sulphate-polyacrylamide gel electrophoresis (SDS-PAGE), followed by processes described in Kolbert et al. [58].

### 2.8. Statistical Analysis

Results are presented as Mean ± S.E. Multiple comparison analyses were completed with SigmaStat 12 software (Systat Software, Inc., San Jose, CA, USA) using investigation of variance (ANOVA; *p* < 0.05) and Duncan’s test. Throughout the experiments several plant generations were cultivated, and all experiments and measurements were carried out at least two times.

## 3. Results and Discussion

### 3.1. Changes in the Ni Content of the Soil and the Plants

**Figure 1:**
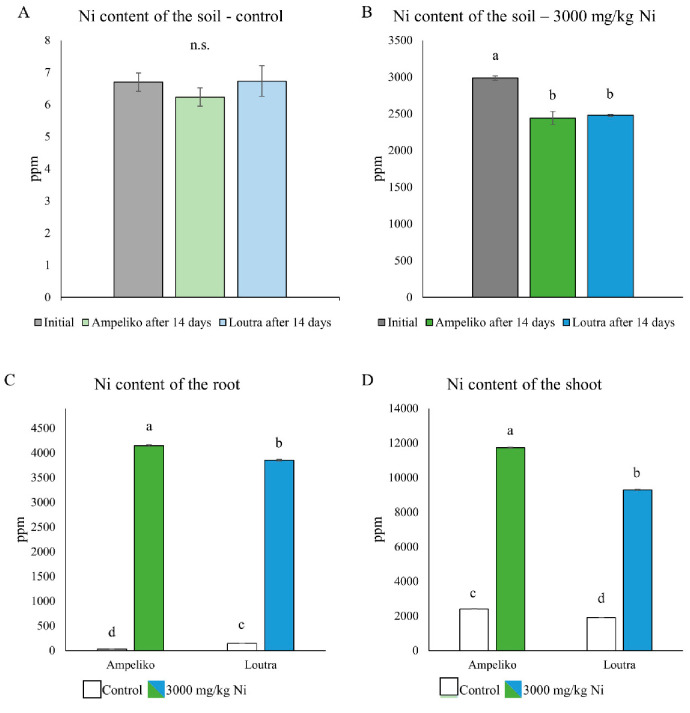
Nickel content of the control soil (**A**) and the soil containing 3000 mg/kg Ni (**B**) before and after 14 days of plant growing in them. Ni content of the roots (**C**) and shoots (**D**) of *O. lesbiaca* from the two different habitats. Different letters indicate significant differences according to Duncan-test (*p* < 0.05), n.s. means no significant difference.

In the present study rhizotrons were filled with two different soil mixtures. Control soil did not contain any added Ni, prior to the experiment it was equilibrated and later irrigated with distilled water. According to the ICP measurements, control soil contained approximately the same amount of Ni in the beginning and at the end of the plants’ growth period (Figure 1A). Ni-treated rhizotrons were filled with a soil mixture in which Ni content was set to 3000 mg/kg (dry weight), with subsequent irrigation with distilled water. During the 14 days-long growth period, *O. lesbiaca* seedlings from both Ampeliko and Loutra sites removed a significant amount of Ni, resulting in 2443 and 2480 ppm residual soil Ni content, respectively (Figure 1B).

Ni content of the plant tissues were also measured, and it is in accordance with the significant depletion of Ni from the soil. Under control circumstances, root tissues of the plants originated from the Loutra site contained significantly more Ni (146 ppm) than plants from the Ampeliko site (33 ppm) (Figure 1C). Regarding the shoots, Ni content showed an opposite tendency in control plants; *O. lesbiaca* plants from the Ampeliko location contained more Ni than plants from the Loutra site (2407 and 1914 ppm, respectively) (Figure 1D). Compared to the concentration of Ni in the soil, the relatively high Ni content of the roots but especially of the shoots can be explained by the high Ni content of the seeds [42,46] and the outer seed coat. *Odontarrhena* species are known to accumulate high amount of Ni in their aerial parts especially in the vacuoles of the epidermal cells and in the base of the non-glandular trichomes [60], thus it is possible the significant amount of Ni contained in the seed was released during germination and taken up by the seedlings.

In the Ni-treated rhizotrons, both the root and shoot tissues of the plants originated from the Ampeliko area contained more Ni—results that are in agreement with those reported by Adamidis et al. [42] under hydroponic conditions. In general, Ni content in both organs was high, especially in the shoots, where plants from Ampeliko accumulated more than 11,000 ppm Ni, several times the base criteria of a hyperaccumulator plant [8].

Another criterion of hyperaccumulator plants is that the bioaccumulation factor (BAF; ratio of Ni concentration in the plant to the soil) needs to be over 1 [53]. Both ecotypes from the Ampeliko and Loutra surpassed this benchmark (6.5 and 5.3, respectively), confirming that *O. lesbiaca* plants from both sites are accumulating a high amount of Ni.

Phytoremediation potential of a plant can be further assessed by the investigation of translocation factor (TF) of a metal, and it is calculated as a shoot to root concentration ratio [61]. TF values were over 1 in both ecotypes, reaching 2.82 in Ampeliko and 2.41 in Loutra, indicating that both ecotypes are effectively translocating Ni into their aerial parts, thus can be used for phytoremediation processes.

It has to be noted, that both BAF and TF values were higher in plants of the Ampeliko ecotype, which seems to be in correlation with the higher soil Ni content of the original habitat, compared to Loutra [19], raising the possibility that the specimens living there adapted to the higher Ni presence and they pass this on to their descendants in some way.

### 3.2. Ni Induced Changes in the Root System Architecture

**Figure 2:**
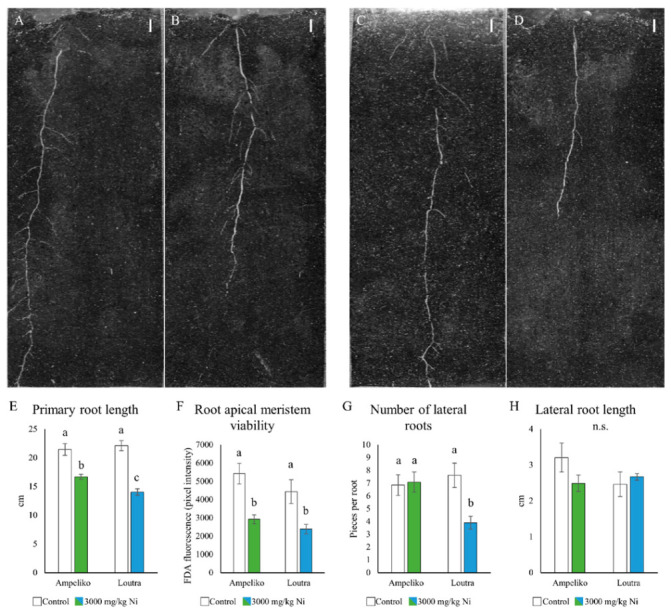
Representative images of the effect of nickel on the root system architecture of 14-days-old *O. lesbiaca* from the two different habitats: (**A**)—Ampeliko control, (**B**)—Ampeliko 3000 mg/kg Ni, (**C**)—Loutra control, (**D**)—Loutra 3000 mg/kg Ni (bar = 1 cm). Length of the primary root (**E**), viability of the root apical meristem (**F**); number (**G**) and length of lateral roots (**H**). Different letters indicate significant differences according to Duncan-test (*p* < 0.05), n.s. means no significant difference.

The high availability and uptake of Ni affected the root growth and development of the two ecotypes differently. Seedlings originated from the more Ni-rich Ampeliko site tolerated the 3000 ppm Ni treatment better than plants from Loutra. PR length decreased in both cases, but the shortening was more pronounced in case of Loutra (Figure 2E). In addition, plants from Ampeliko proved to be more tolerant in terms of LR number: Ni treatment did not inhibit lateral root formation, while in plants from Loutra had significantly less lateral roots under Ni stress (Figure 2G). Interestingly, the difference between the two ecotypes in their relative Ni tolerance did not appear in the viability of the root apical meristems (Figure 2F); Ni treatment decreased it in both cases. In terms of the length of the lateral roots, there were no significant difference between the two ecotypes; however, it has to be noted that while the number of the lateral roots did not decrease in plants from Ampeliko, their length reduced slightly (Figure 2H). For comparison, the root growth response to Ni of *Brassica napus* was also examined. As a contrast, its root growth was significantly inhibited by Ni in a much lower concentration: approximately 160 ppm Ni caused 50% growth inhibition, while 200 ppm almost inhibited root growth completely (Figure S2).

Orientation of the lateral roots might be in connection with the degree of stress and the tolerance of the plant against it [39,49]. In the present experimental setup, in the case of the more tolerant *O. lesbiaca* plants from the Ampeliko site, the angle of the lateral roots enclosed with the primary root changed to the horizontal direction (from 52° to 57°; Figure S3), while in the more sensitive plants from the Loutra site showed no such response (remained around 55°). In previous studies, we found that zinc (Zn) in a growth-promoting amount also resulted in a more horizontal orientation, while Zn in a growth inhibiting concentration induced a more vertical lateral root alignment [49]. In this case, the re-orientation of the lateral roots to a more horizontal direction seems to support the relative Ni tolerance of plants originated from the Ampeliko site compared to Loutra. However, it has to be noted that changes in the lateral root angle due to heavy metal stress seem to be species-specific, since the root growth of sunflower (*Helianthus annuus*) was significantly inhibited by combined heavy metal treatment, but the orientation of its lateral roots changed to a more horizontal direction as well [39].

### 3.3. Differences in the Underlying Nitro-Oxidative Status

In the background of the different root growth response the nitro-oxidative homeostasis showed distinct changes in the seedling originated from the two different sites.

**Figure 3:**
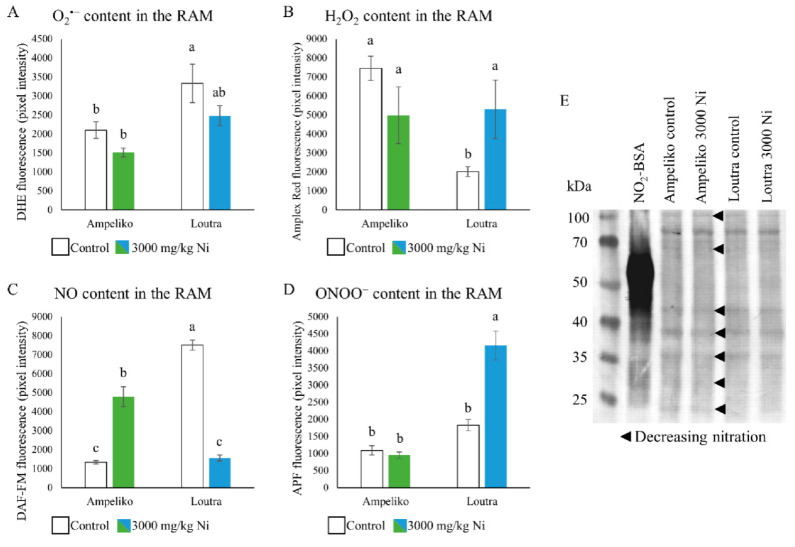
Changes in the fluorescence consistent with superoxide (**A**), hydrogen peroxide (**B**), nitric oxide (**C**) and peroxynitrite (**D**) in the root apical meristem (RAM) of *O. lesbiaca* from the two different habitats. Representative immunoblot showing nickel-induced changes of protein tyrosine nitration in the roots of *O. lesbiaca* from the two different habitats (**E**). Arrows show lowered nitration compared to the control. Different letters indicate significant differences according to Duncan-test (*p* < 0.05), n.s. means no significant difference.

In the relatively tolerant plants from the Ampeliko site, O_2_^•−^-dependent fluorescence was generally lower and did only decrease in a non-significant manner as the effect of Ni exposure. On the other hand, in the root tips of the more sensitive plants from Loutra, fluorescence consistent with O_2_^•−^ was higher under control circumstances but decreased due to the applied Ni treatment (Figure 3A). Ni exposure only slightly decreased the H_2_O_2_ linked fluorescence again in root tips of the Ampeliko ecotype, and while it was significantly lower in the Loutra ecotype under control circumstances, Ni treatment increased the H_2_O_2_ content of their root tips significantly (Figure 3B).

Fluorescence consistent with the NO content of the root tips changed in an opposite direction in the two ecotypes. In the Ampeliko ecotype, the low content increased significantly under Ni stress, while the high NO content detected in control root tips of the Loutra ecotype decreased significantly upon exposure to 3000 ppm Ni (Figure 3C). Excess Ni did not induce significant change in the ONOO^−^-linked fluorescence in the root tips of the relatively more tolerant plants from Ampeliko; however, it greatly increased ONOO^-^ level in the sensitive Loutra ecotype (Figure 3D).

The balance of the nitro-oxidative signaling network was evidently less affected by excess Ni in the more tolerant Ampeliko ecotype, since only the level of NO increased significantly. In the relatively sensitive plants from the Loutra site, all the investigated elements of the above-mentioned network suffered Ni-induced changes. Levels of O_2_^•−^ and NO decreased, while their reaction product, ONOO^−^ was being accumulated in the root tips. Interestingly, this ONOO^−^ accumulation was not accompanied by the intensification of protein tyrosine nitration. Moreover, in the root system of the Ampeliko ecotype, protein tyrosine nitration decreased as the effect of Ni exposure compared to healthy plants (Figure 3E).

Relatively little is known about the relationship between excess Ni, tolerance mechanisms and the balance of RNS in plants so far. In rice (*Oryza sativa*), exogenously increased NO content of the root was linked with the plant’s better Ni tolerance [62]. In bean (*Phaseolus vulgaris*), NO donor sodium nitroprusside likewise successfully attenuated oxidative stress triggered by Ni exposure [63]. In addition, in finger millet (*Eleusine coracana*) exogenous application of NO (especially together with salicilic acid) was also able to reduce the toxic effect of excess Ni [64]. These findings can be set in parallel with the Ni-induced NO accumulation observed in the root tips, coupled with the relative higher tolerance of the Ampeliko ecotype, compared to Loutra in our experiments.

Similarly to our results, where the responses of *Arabidopsis thaliana* and *Brassica juncea* to Ni was compared, the more tolerant *B. juncea* was able to avoid changes in its nitro-oxidative homeostasis and protein tyrosine nitration increased in a smaller degree, compared to the Ni-sensitive *A. thaliana* [29]. In the present study, the relative tolerance of the two ecotypes and the degree of protein tyrosine nitration shows an analogous connection, as in the more tolerant Ampeliko ecotype, strength of the immunopositive bands decreased in the proteome of Ni treated roots, while the relative sensitivity of the Loutra ecotype was joined with unchanged nitration response. Moreover, in the selenium hyperaccumulator serpentine plant *Astragalus bisulcatus*, the degree of protein tyrosine nitration also decreased under selenium stress, compared to the sensitive *A. membranaceus*, where it increased significantly [58]. The degradation of the proteins, including nitrated ones resulting in the observed low detectability of nitrated proteins might act as a tolerance mechanism against Ni stress. The exact mechanism behind this is yet to be discovered, but protein decomposition via proteosomes can serve as a possible explanation [58,65,66].

## 4. Conclusions

Excess nickel affected the root architecture of the two *O. lesbiaca* ecotypes differently. Plants from both habitats met the requirements of a hyperaccumulator plant and were able to accumulate and translocate a high amount of Ni; however, the Ampeliko ecotype proved to be more effective. Plants from the Ampeliko site also demonstrated higher tolerance, since their root growth was less inhibited by excess Ni, possibly because Ni content of the soil at the Ampeliko site is considerably higher than at the Loutra one, suggesting that the specimens living there successfully adapted to the higher Ni presence. In the background of the different root growth responses, the nitro-oxidative homeostasis shown distinct changes. In the root tips of the more tolerant Ampeliko ecotype, NO was being accumulated, while the degree of protein tyrosine nitration decreased. These result suggest, on the one hand, that in this case NO acted as a signaling molecule (towards tolerance mechanisms), not a stress indicator, and on the other hand, that the decrease in protein tyrosine nitration may indicate this ecotype’s better relative tolerance compared to Loutra’s. In the relatively less tolerant Loutra ecotype, NO content of the root tips decreased significantly, further suggesting that its elevated level might contribute to the better Ni tolerance of a plant.

Our results suggest that Ni hypertolerance and hyperaccumulation capacity might be connected to the ability of a plant species to maintain its RNS balance and to reduce the sensitivity of the proteome to nitration. Moreover, the fact that the descendants of the two ecotypes have different sensitivity to excess Ni, raises the possibility that such properties of a species’ might be influenced by their living space and they can be genetically inherited.

## Supplementary Materials

The following are available online at https://www.mdpi.com/2076-3921/9/9/837/s1. Figure S1. Representative root system architecture images showing the effect of different concentrations of Ni on the two ecotypes of *O. lesbiaca*, compared to the control (bar = 1 cm). Figure S2. Length of the primary root and number of the lateral roots of rapeseed (*Brassica napus*) subjected to different concentrations of Ni. Figure S3. Visualization of the changes in the angle of the lateral roots with the primary root in the more tolerant *O. lesbiaca* plants from the Ampeliko site.
