# Supplementary figures and images for: Different Nitro-Oxidative Response of Odontarrhena lesbiaca Plants from Geographically Separated Habitats to Excess Nickel
Source: Antioxidants (Basel). 2020 Sep 7;9(9):837. doi: 10.3390/antiox9090837 (PMC7554898; doi:10.3390/antiox9090837)

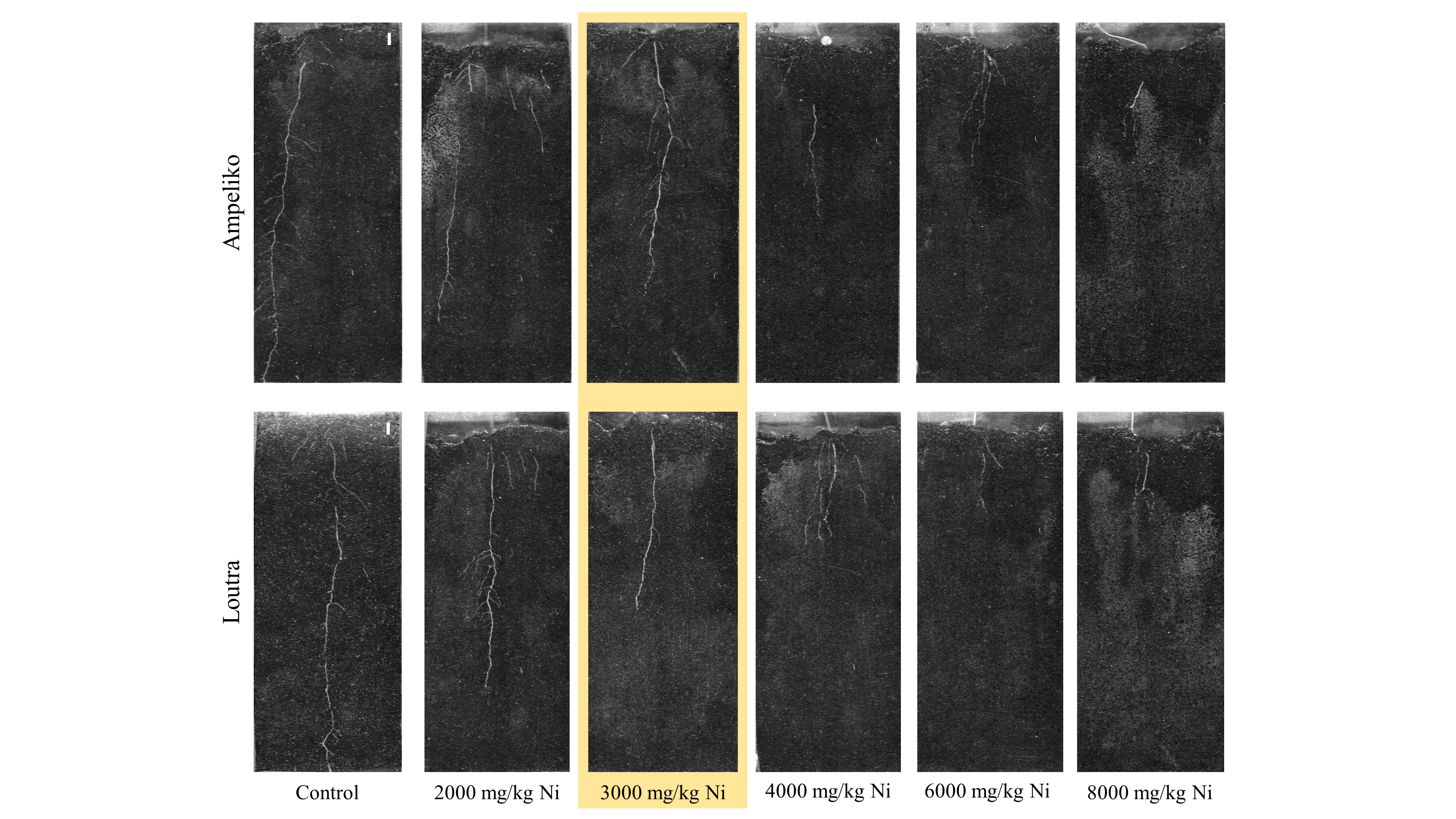

Supplement: Supplementary file 1 [file antioxidants-09-00837-s001.zip › SupplFig1.tif]

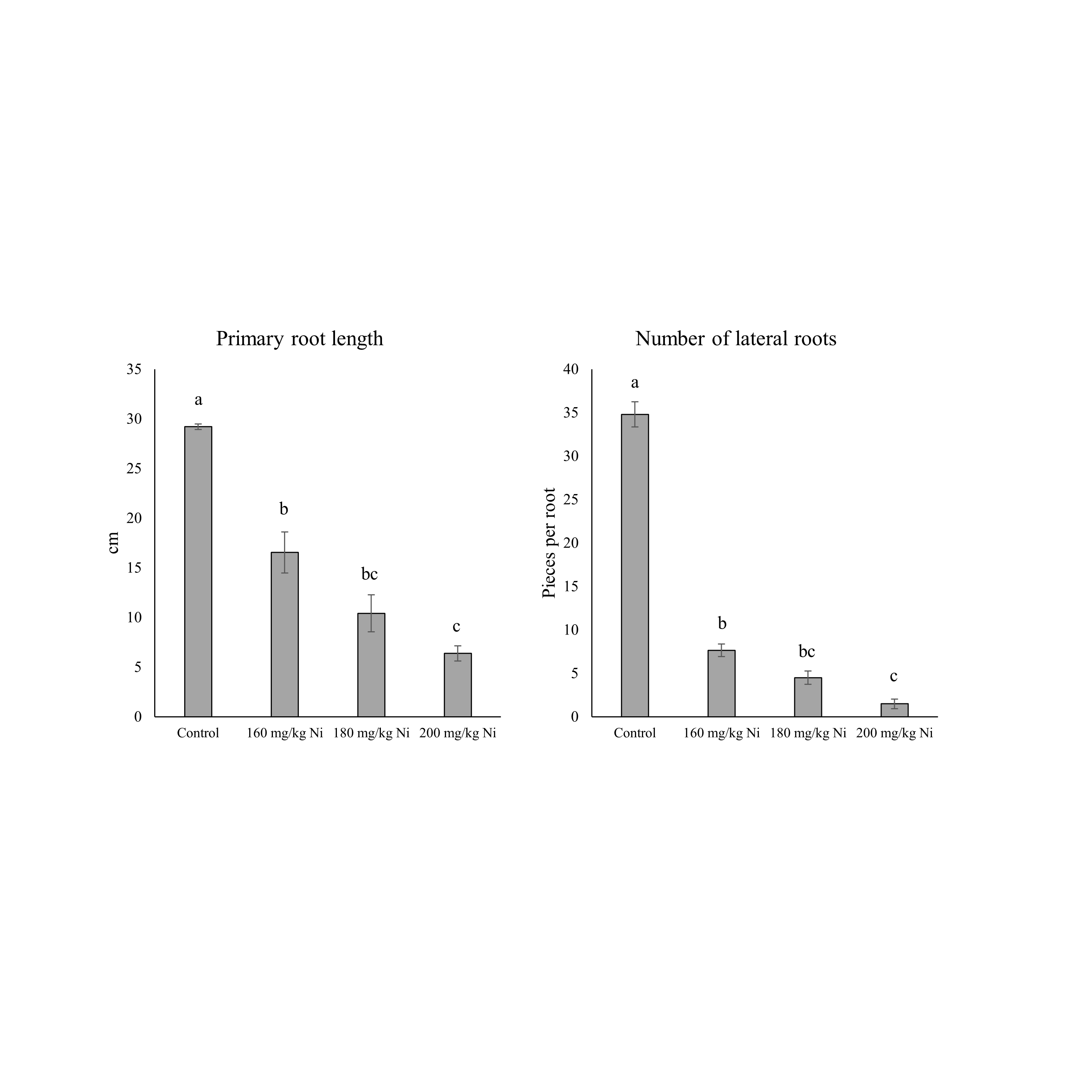

Supplement: Supplementary file 1 [file antioxidants-09-00837-s001.zip › SupplFig2.tif]

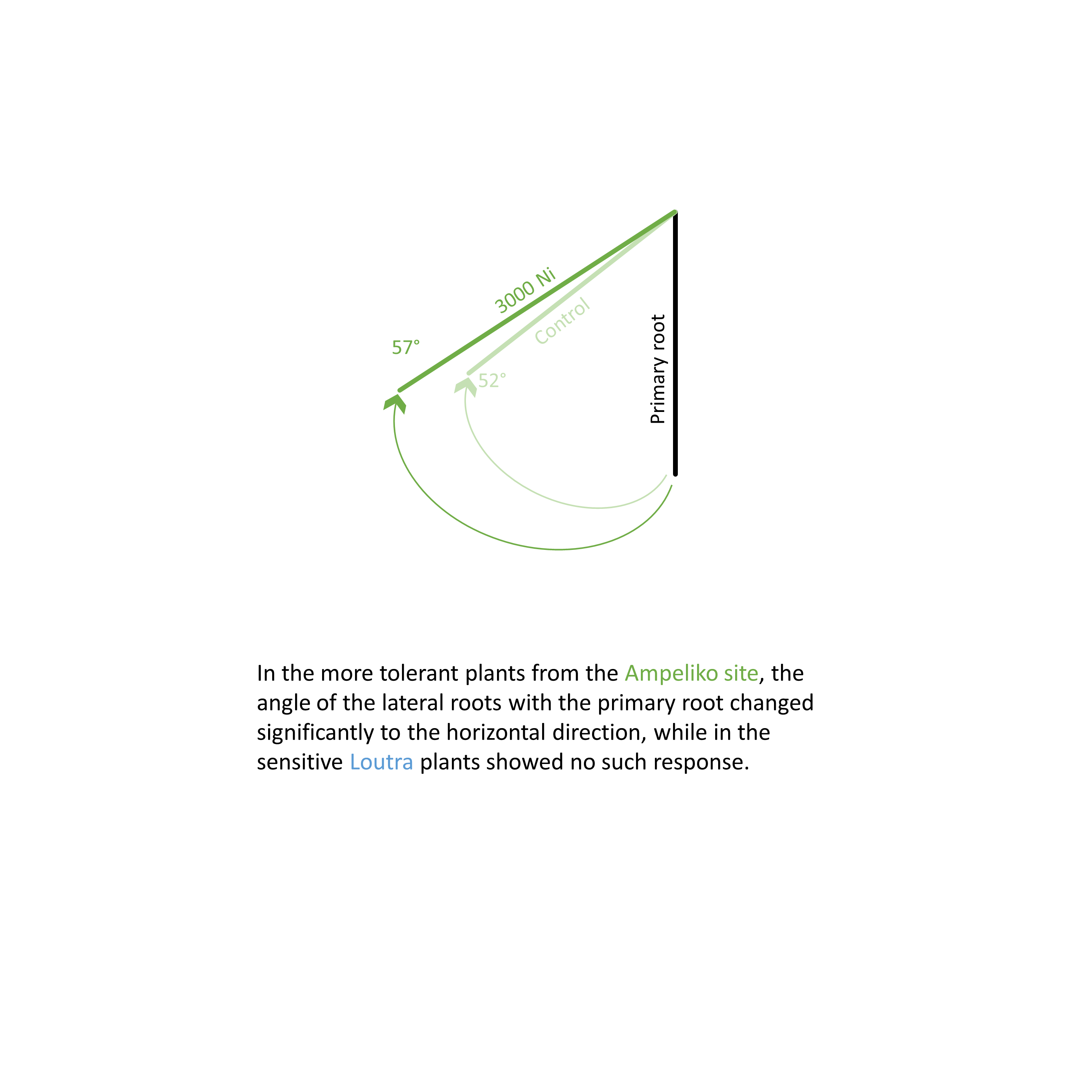

Supplement: Supplementary file 1 [file antioxidants-09-00837-s001.zip › SupplFig3.TIF]
